# Supplementary material for: Adenosine/β-Cyclodextrin-Based Metal–Organic Frameworks as a Potential Material for Cancer Therapy
Source: Biomolecules. 2023 Jul 20;13(7):1154. doi: 10.3390/biom13071154 (PMC10377648; doi:10.3390/biom13071154)
Supplement: Supplementary file 1 [file biomolecules-13-01154-s001.zip › biomolecules-2494853-supplementary.pdf]

Adenosine:β-cyclodextrin based metal-organic frameworks as a potential material for cancer therapy

Rajaram Rajamohan[a]*, Sekar Ashokkumar[b], Mani Murali Krishnan[c], Kuppusamy Murugavel[d], Moorthiraman Murugan[e], and Yong Rok Lee[a]*

[a] School of Chemical Engineering, Yeungnam University, Gyeongsan 38541, Republic of Korea.

[b] Plasma Bioscience Research Center, Department of Electrical and Biological Physics, Kwangwoon University, Seoul 01897, Republic of Korea.

[c] Department of Chemistry, Bannari Amman Institute of Technology, Sathyamangalam - 638 401, Tamil Nadu, India.

[d] PG and Research Department of Chemistry, Government Arts College, Chidambaram - 608 102, Tamil Nadu, India.

[e] Department of Chemistry, IFET College of Engineering, Villupuram - 605 108, Tamil Nadu, India.

*Correspondence: rajmohanau@yu.ac.kr (R.R.); yrlee@yu.ac.kr (Y.R.L.)

Preparation of β-CD-K MOFs

To synthesize β-CD-K MOFs, an improved methanol vapor diffusion method is employed. The process involved dissolving 1030 mg of β-CD in 300 ml of deionized water and 696 mg of KH_2PO_4 in 10 ml of deionized water, creating an aqueous solution. The solution was then stirred for 30 minutes and filtered through a 0.45 μm membrane into a glass vessel. To facilitate the methanol diffusion process, 0.5 ml of methanol was added to the vessel, which was then sealed in a beaker containing methanol (MeOH). The entire solution was left undisturbed for 10 days, allowing for methanol diffusion facilitated by the surrounding 40 ml of methanol. As a result, colorless crystals were formed. These crystals were subsequently washed three times with isopropanol and subjected to solvent exchange by soaking them in dichloromethane (CH_2Cl_2) for three days. After vacuum drying the sample overnight at 40 °C, the final product was named β-CD-K MOFs.

***In-vitro* ADN release from ADN:β-CD-K MOFs**

The release of ADN from a polymer matrix was investigated by conducting a kinetic study over time at room temperature. The study was carried out using phosphate buffer solutions with pH values of 5.0, 6.5, and 7.8. A 10 mg/5 mL polymer-drug matrix was prepared and placed within a dialysis membrane tube with a molecular cut-off of 12000 Da (Himedia, Dialysis membrane-110). This tube was immersed in a beaker containing 30 mL of PBS buffer. At different time intervals, 2 mL of the release medium was withdrawn and replaced with fresh PBS buffer. The amount of ADN released was determined by measuring the absorbance of the release medium using a UV-Visible spectrophotometer at a wavelength of 260 nm. This process allowed for the monitoring of the release kinetics of ADN from the polymer matrix. The experiments were conducted in triplicate to ensure reliable and consistent results.

Lungs fibroblasts MRC-5 and triple-negative breast cancer MB 231 cell culture

The MRC-5 and MDA-MB-231 cells are cultured in a T-75 flask containing Dulbecco’s Modified Eagle Medium (DMEM) supplemented with 10% fetal bovine serum and 1% antibiotic (streptomycin & penicillin). The cells inoculated plates are incubated at 37 °C in 5% CO_2 for up to 2 days, and the cells reached ~80% confluence to start the cell viability experiment.

Cytotoxicity (*in-vitro* cell viability)

The *in-vitro* cell viability of the ADN, β-CD-K MOFs, and ADN:β-CD-K MOFs are investigated by Alamar blue assay. Briefly, MRC-5 and MDA-MB-231 cells are seeded into 96 well plates at a density of 2×10^4 cells/well when cells reached 80% confluence in 1 day. Then, the growth medium (DMEM) was removed and one time washed with PBS. After PBS washing, 200 µl of fresh medium (DMEM) containing different concentrations of derivatives (0.2, 2, 20, 200, and 2000 µg/ml) is added into each well and incubated for up to 3 days. Based on diverse instance periods, the cells are washed with PBS and further added 100 µl of 10% of Alamar blue, incubated at 37 °C with 5% CO_2 for up to 4 hours. After incubation plate was read at excitation and emission wavelength of 540 and 600 nm respectively using a microplate reader Synergy HT spectrophotometer (BioTek, Winooski, VT, USA).

Characterization Techniques

Various techniques are used to analyze the MOFs, including FT-IR spectroscopy, Raman spectroscopy, proton NMR, FE-SEM with EDX spectroscopy, XRD, XPS, and DSC. FT-IR spectra are recorded with the Perkin Elmer Spectrum Two in transmittance mode within the ranges of 400.0 - 4000.0 cm^{-1}. 16 scans for the measurement at a resolution of 8 cm^{-1}. Raman spectral measurements are captured on the XploRA Micro-Raman spectrophotometer (Horiba) in the 100.0 - 4000.0 cm^{-1} range. Proton nuclear magnetic resonance (^{1}H NMR) spectroscopy (Bruker NMR spectrometer with 600.0 MHz) with DMSO-d6 solvent. At a 10.0 kV accelerating voltage, FE-SEM, and EDX spectral analysis is carried out with the Hitachi S-4800. Powder XRD measurements are performed on a PANalytical X'Pert3 MRD diffractometer at 40.0 kV and 30 mA with monochromatized Cu K radiation (λ =1.54 Å). The 2θ range is 10° - 80° at a scan rate of 5° min^{-1} and a wavelength of 1.5405 Å. K-Alpha is used to generate XPS spectra (Thermo Scientific). The CasaXPS software S3 is used to deconvolve the high-resolution XPS spectra. The thermal behavior of the MOFs is performed using TA instruments, and the curves are of individual elements analyzed using the Universal V4.5A Program. The weight of each sample is around 3.5 mg and the temperature range for the measurement is about 40.0 – 250 °C under a dynamic nitrogen atmosphere (50 mL min−1) with increments of 10 °C/minute. All of the above instrument services are utilized at the core research support center (CRSC) for natural products and medical materials at Yeungnam University, South Korea.

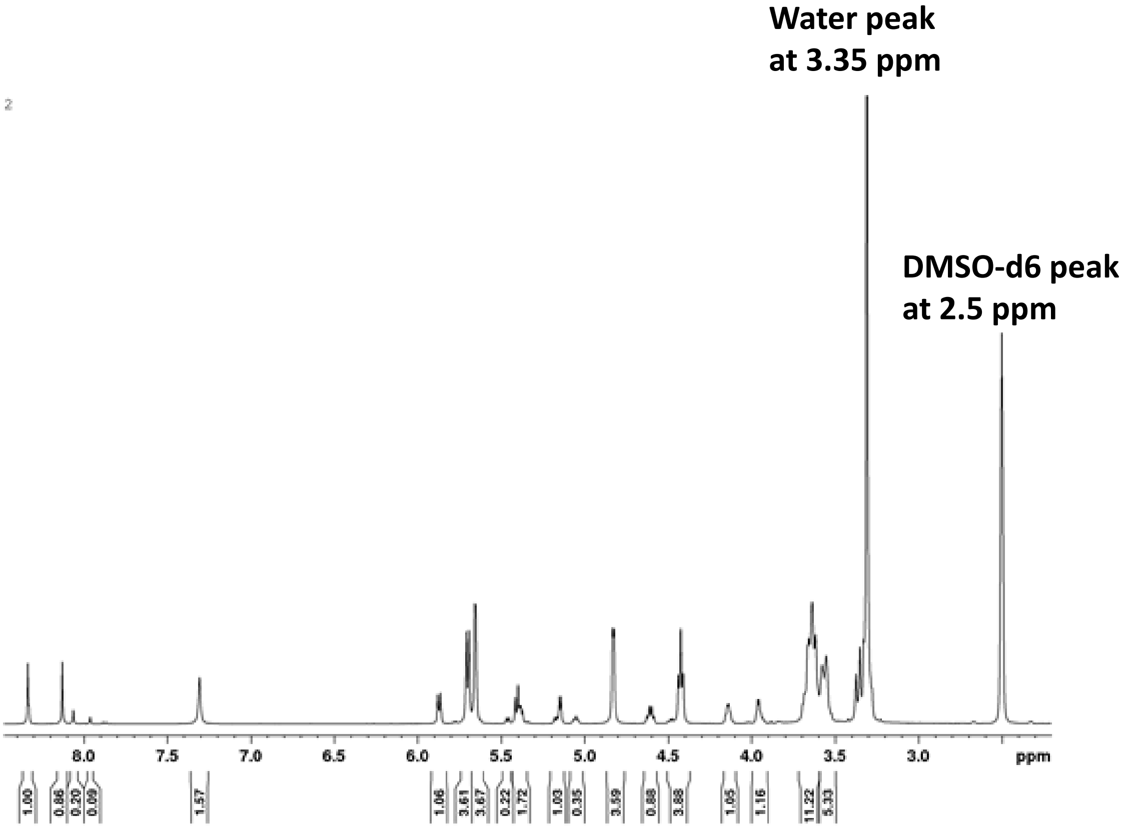

Figure S1. ^{1}H NMR spectra of ADN:β-CD inclusion complex (Bruker NMR spectrometer with 400.0 MHz with DMSO-d6 solvent)

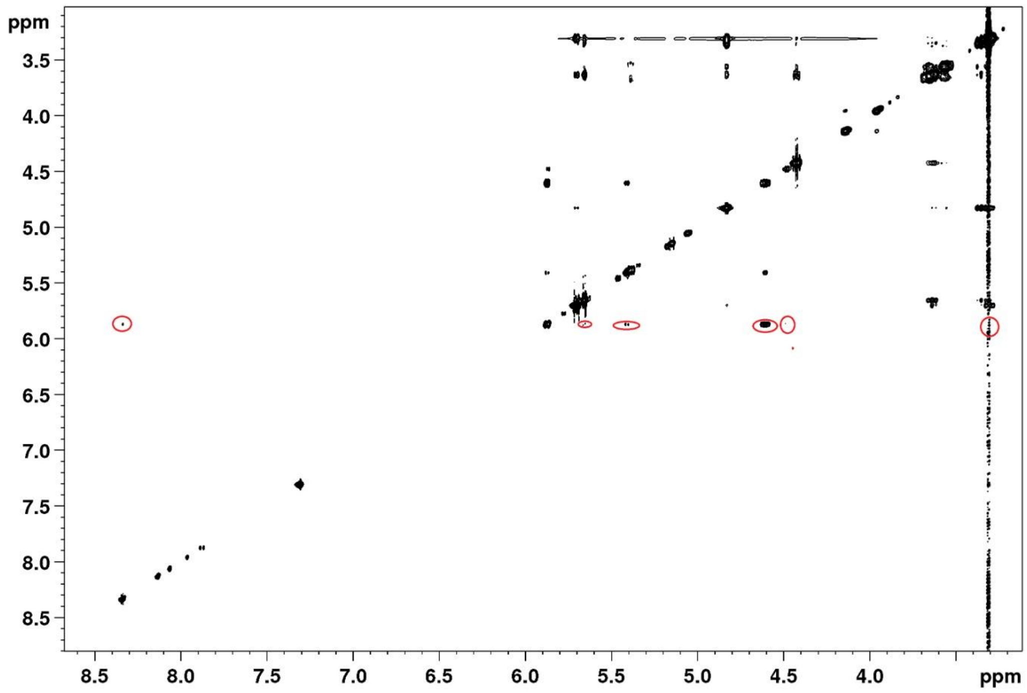

Figure S2. ROESY of ADN:β-CD Inclusion complex.

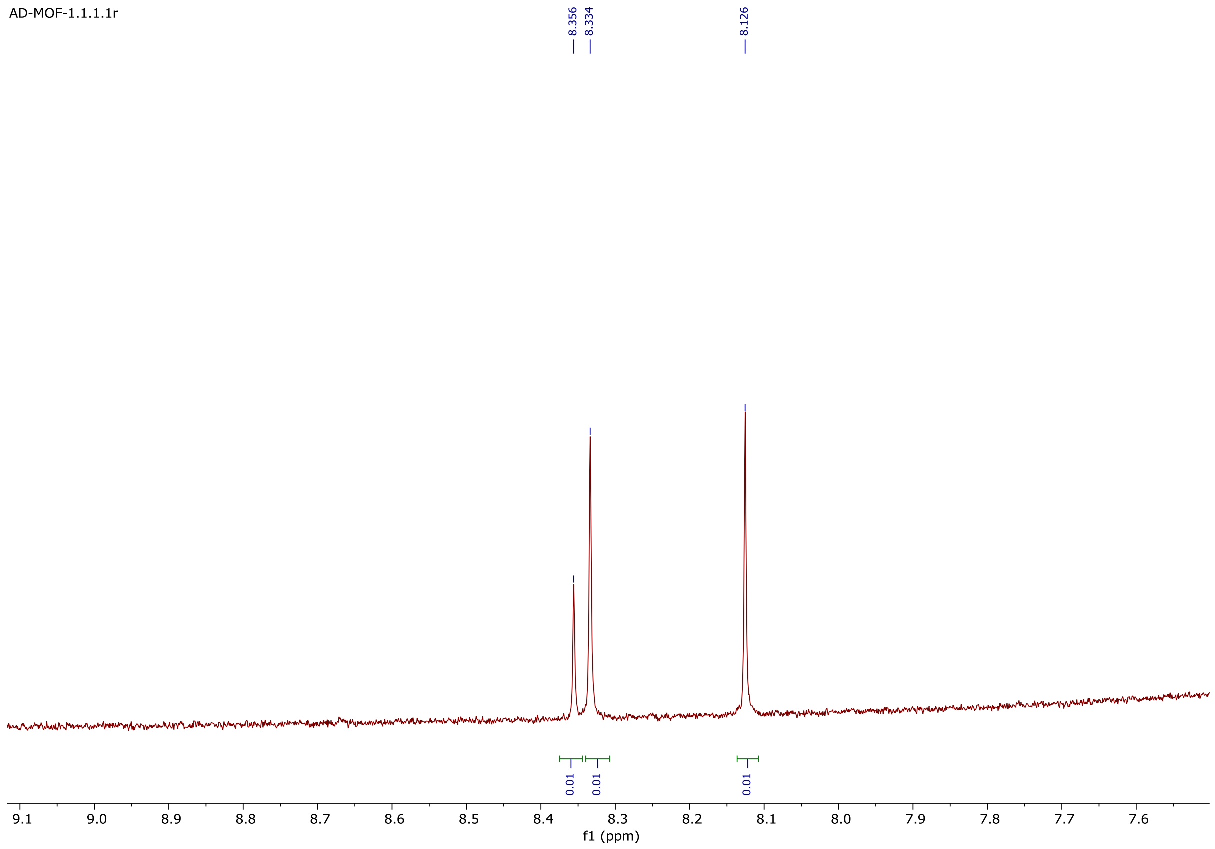

Figure S3a. ^{1}H NMR spectra of ADN:β-CD-K MOFs (expanded).

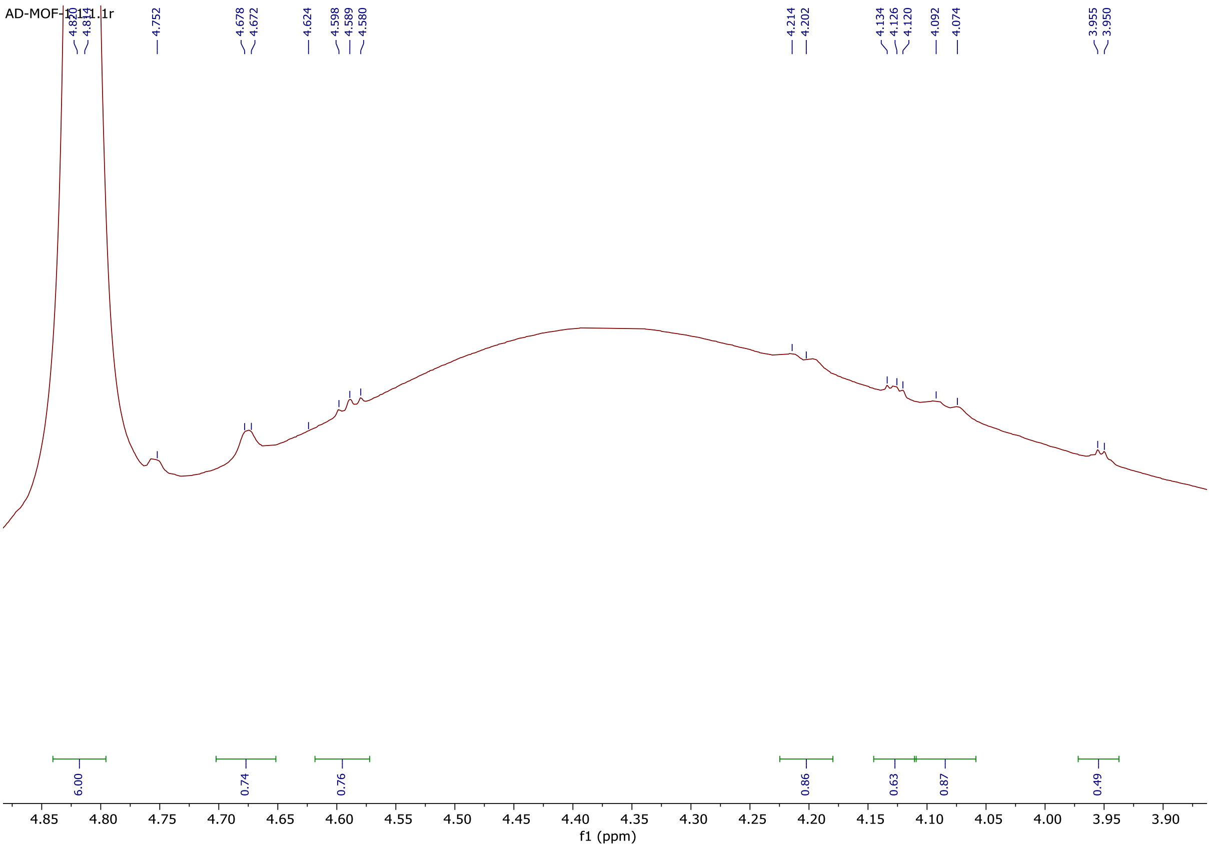

Figure S3b. ^{1}H NMR spectra of ADN:β-CD-K MOFs (expanded).

Table S1. The BE values and atoms in percentages of both MOFs.

Name of the samples	C 1s		O 1s		K 2p		N 1s	
	BE (eV)	Atomic %	BE (eV)	Atomic %	BE (eV)	Atomic %	BE (eV)	Atomic %
**β-CD-K MOFs**	285.68	55.04	531.98	42.89	292.18	2.07	-	-
**ADN:β-CD-K MOFs**	285.68	55.06	531.98	42.87	292.38	0.85	398.78	0.72

*Binding energy is denoted as BE.

Table S2. Proton Chemical Shifts of β-CD-K MOFs, ADN, and ADN:β-CD-K MOFs

Positions	Chemical shifts (ppm)			Assignment of multiplicities
	β-CD-K MOFs	ADN	ADN:β-CD-K MOFs	
OH protons at equatorial positions	4.82, 5.67	-	-	Doublet of doublet
Methine proton axial (CH_2OH attached proton)	4.45	-	-	Triplet
CH and CH_2 protons	3.30, 3.56, and 3.63	-	-	Multiplet
OH of CH_2OH	3.33	-	-	Multiplet
Equatorial hydrogen	5.73	-	-	Doublet
H-2	-	8.35	-	Singlet
H-8	-	8.14	-	Singlet
NH2 at C-6	-	7.36	-	Broad singlet
OH at C-2'	-	5.46	-	Doublet (4 Hz)
OH at C-3'	-	5.25	-	Doublet (4 Hz)
OH at C-5' (attached to methylene carbon)	-	3.96-3.98	-	Multiplet
H-1'	-	5.88	-	Doublet (4 Hz)
H-2'	-	4.60-4.63	-	Multiplet
H-3'	-	4.14-4.16	-	Multiplet
H-4'	-	5.43-5.45	-	Multiplet
H-5' (methylene protons)	-	3.54-3.58, 3.66-3.69	-	Multiplet
H-2	-	-	8.35	Singlet
H-8	-	-	8.33	Singlet

NH_2 at C-6	-	-	8.12	Singlet
OH at C-2'	-	-	4.95	Broad singlet
OH at C-3'	-	-	4.75	Broad singlet
OH at C-5' (attached to methylene carbon)	-	-	3.23	Multiplet
H-1'	-	-	5.85	Doublet (4 Hz)
H-2'	-	-	4.67 bs	Broad singlet
H-3'	-	-	4.20	Doublet (4.8 Hz)
H-4'	-	-	4.59	Multiplet
H-5' (methylene protons)	-	-	4.08, and 3.72,	Multiplet
OH protons at equatorial positions	-	-	4.82	Singlet
Methine proton axial (CH_2OH attached proton)	-	-	3.65-5.53	Multiplet
CH and CH_2 protons	-	-	3.28-3.51, and 3.53-3.58	Multiplet.
OH of CH_2OH	-	-	-	-
Equatorial hydrogen	-	-	3.65-5.53	Multiplet

Table S3. The cytotoxic ability of ADN, and ADN:β-CD-K MOFs on cancer cell lines.

Name of the sample	Concentration (μg/ml)	MRC-5				MDA-MB-231			
		Day 1		Day 3		Day 1		Day 3	
		% of survival	SD	% of survival	SD	% of survival	SD	% of survival	SD
	Control	100.00	0	100.00	0	100.00	0	100.00	0
ADN	0.2	96.93	0.24	97.84	1.21	97.86	1.38	90.53	0.15
	2	94.42	0.03	96.37	0.03	96.78	0.37	88.14	0.48
	20	92.42	0.22	96.44	0.37	95.11	0.05	87.04	0.79
	200	91.22	0.21	94.65	0.98	94.05	1.10	78.57	0.61
	2000	83.23	0.84	85.65	0.52	87.95	0.48	72.28	0.28
ADN:β-CD-K MOFs	Control	100.00	0	100.00	0	100.00	0	100.00	0
	0.2	98.7	0.77	99.1	0.28	98.3	0.39	97.8	0.17
	2	93.9	0.24	97.7	0.27	96.1	0.71	96.0	0.82
	20	91.2	0.49	97.5	0.14	95.1	0.06	94.4	0.01
	200	91.0	0.36	97.2	0.27	94.5	0.00	94.2	0.18
	2000	90.6	0.36	95.9	0.14	91.4	0.00	91.3	0.00

*SD : Standard Deviation
